# The effect of Alzheimer's disease genetic factors on limbic white matter microstructure

**DOI:** 10.1002/alz.70130

**Published:** 2025-04-12

**Authors:** Anna Lorenz, Aditi Sathe, Dimitrios Zaras, Yisu Yang, Alaina Durant, Michael E. Kim, Chenyu Gao, Nancy R. Newlin, Karthik Ramadass, Praitayini Kanakaraj, Nazirah Mohd Khairi, Zhiyuan Li, Tianyuan Yao, Yuankai Huo, Logan Dumitrescu, Niranjana Shashikumar, Kimberly R. Pechman, Trevor Bryan Jackson, Abigail W. Workmeister, Shannon L. Risacher, Lori L. Beason‐Held, Yang An, Konstantinos Arfanakis, Guray Erus, Christos Davatzikos, Mohamad Habes, Di Wang, Duygu Tosun, Arthur W. Toga, Paul M. Thompson, Elizabeth C. Mormino, Panpan Zhang, Kurt Schilling, Alzheimer's Disease Neuroimaging Initiative (ADNI)The BIOCARD Study TeamThe Alzheimer's Disease Sequencing Project (ADSP), Marilyn Albert, Walter Kukull, Sarah A. Biber, Bennett A. Landman, Sterling C. Johnson, Barbara Bendlin, Julie Schneider, Lisa L. Barnes, David A. Bennett, Angela L. Jefferson, Susan M. Resnick, Andrew J. Saykin, Timothy J. Hohman, Derek B. Archer

**Affiliations:** ^1^ Vanderbilt Memory and Alzheimer's Center Vanderbilt University School of Medicine Nashville Tennessee USA; ^2^ Vanderbilt Brain Institute Vanderbilt University Medical Center Nashville Tennessee USA; ^3^ Department of Computer Science Vanderbilt University Nashville Tennessee USA; ^4^ Department of Electrical and Computer Engineering Vanderbilt University Nashville Tennessee USA; ^5^ Department of Neurology Vanderbilt University Medical Center Nashville Tennessee USA; ^6^ Vanderbilt Genetics Institute Vanderbilt University Medical Center Nashville Tennessee USA; ^7^ Department of Radiology and Imaging Sciences Indiana University School of Medicine Indianapolis Indiana USA; ^8^ Indiana Alzheimer's Disease Research Center Indiana University School of Medicine Indianapolis Indiana USA; ^9^ Laboratory for Behavioral Neuroscience National Institute on Aging, National Institutes of Health Baltimore Maryland USA; ^10^ Department of Biomedical Engineering Illinois Institute of Technology Chicago Illinois USA; ^11^ Rush Alzheimer's Disease Center Rush University Medical Center Chicago Illinois USA; ^12^ Department of Diagnostic Radiology Rush University Medical Center Chicago Illinois USA; ^13^ Department of Radiology University of Pennsylvania Philadelphia Pennsylvania USA; ^14^ Glenn Biggs Institute for Alzheimer's and Neurodegenerative Diseases University of Texas Health Science Center at San Antonio San Antonio Texas USA; ^15^ University of Texas Health Science Center at San Antonio San Antonio Texas USA; ^16^ Department of Radiology and Biomedical Imaging University of California San Francisco San Francisco California USA; ^17^ Laboratory of Neuroimaging USC Stevens Institute of Neuroimaging and Informatics Keck School of Medicine University of Southern California Los Angeles California USA; ^18^ Imaging Genetics Center Mark and Mary Stevens Institute for Neuroimaging and Informatics Keck School of Medicine University of Southern California Marina del Rey California USA; ^19^ Department of Neurology and Neurological Sciences Stanford University School of Medicine Stanford California USA; ^20^ Department of Biostatistics Vanderbilt University Medical Center Nashville Tennessee USA; ^21^ Department of Radiology & Radiological Sciences Vanderbilt University Medical Center Nashville Tennessee USA; ^22^ Vanderbilt University Institute of Imaging Science Vanderbilt University Medical Center Nashville Tennessee USA; ^23^ Department of Neurology Johns Hopkins School of Medicine Baltimore Maryland USA; ^24^ National Alzheimer's Coordinating Center University of Washington Seattle Washington USA; ^25^ Department of Biomedical Engineering Vanderbilt University Nashville Tennessee USA; ^26^ Wisconsin Alzheimer's Disease Research Center School of Medicine and Public Health University of Wisconsin Madison Wisconsin USA; ^27^ Wisconsin Alzheimer's Institute School of Medicine and Public Health University of Wisconsin Madison Wisconsin USA

**Keywords:** Alzheimer's disease, diffusion MRI, free water, genetic risk variants, polygenic risk, white matter microstructure

## Abstract

**INTRODUCTION:**

White matter (WM) microstructure is essential for brain function but deteriorates with age and in neurodegenerative conditions such as Alzheimer's disease (AD). Diffusion MRI, enhanced by advanced bi‐tensor models accounting for free water (FW), enables in vivo quantification of WM microstructural differences.

**METHODS:**

To evaluate how AD genetic risk factors affect limbic WM microstructure – crucial for memory and early impacted in disease – we conducted linear regression analyses in a cohort of 2,614 non‐Hispanic White aging adults (aged 50.12 to 100.85 years). The study evaluated 36 AD risk variants across 26 genes, the association between AD polygenic scores (PGSs) and WM metrics, and interactions with cognitive status.

**RESULTS:**

AD PGSs, variants in *TMEM106B*, *PTK2B*, *WNT3*, and apolipoprotein E (*APOE*), and interactions involving *MS4A6A* were significantly linked to WM microstructure.

**DISCUSSION:**

These findings implicate AD‐related genetic factors related to neurodevelopment (*WNT3*), lipid metabolism (*APOE*), and inflammation (*TMEM106B*, *PTK2B, MS4A6A*) that contribute to alternations in WM microstructure in older adults.

**Highlights:**

- AD risk variants in *TMEM106B*, *PTK2B*, *WNT3*, and *APOE* genes showed distinct associations with limbic FW‐corrected WM microstructure metrics.
- Interaction effects were observed between *MS4A6A* variants and cognitive status.
- PGS for AD was associated with higher FW content in the limbic system.

## 1 BACKGROUND

White matter (WM) microstructure, comprising myelinated axons that facilitate efficient signal transmission between neurons, is crucial for brain function, but it deteriorates with age and is significantly affected in Alzheimer's disease (AD).^1^, ^2^, ^3^ The WM microstructure, which connects medial temporal lobe structures with parietal and fontal cortices, is particularly important for memory function, and its deterioration is integral to the pathophysiological changes observed in AD.^4^, ^5^ Notably, WM alterations can be detected in the preclinical stages of AD pathology, preceding changes in hippocampal microstructure.^6^ Despite this, the processes underlying WM degeneration and the molecular pathways leading to these changes remain poorly understood.

Diffusion magnetic resonance imaging (dMRI) allows for the in vivo assessment of these microstructural changes non‐invasively by measuring the diffusion of water molecules in the brain.^7^, ^8^, ^9^, ^10^ This method estimates WM microstructure by detecting the magnitude and directionality of diffusivity within each voxel and derives conventional diffusion tensor imaging (DTI) metrics such as fractional anisotropy (FA_CONV_), mean diffusivity (MD_CONV_), axial diffusivity (AxD_CONV_), and radial diffusivity (RD_CONV_).^11^ FA_CONV_ measures the directional dependence of water diffusion, with higher values typically indicating well‐organized WM tracts. MD_CONV_ represents the average diffusion rate, providing a general measure of tissue density and cellularity. AxD_CONV_ measures diffusion along the primary axis of fibers, with lower values indicating axonal damage. RD_CONV_ assesses diffusion perpendicular to the primary axis, with higher values suggesting demyelination. However, these conventional metrics rely on single tensor models, which are limited by partial volume effects of brain tissue with cerebrospinal fluid. Recent advancements address these limitations and have introduced bi‐tensor models that estimate and correct DTI metrics for free water (FW) content in each voxel.^12^ This approach allows separation between extracellular (FW) and intracellular space (FA_FWcorr_, AxD_FWcorr_, MD_FWcorr_, RD_FWcorr_), providing a more detailed and biologically accurate quantification of WM.^2^, ^3^ This bi‐tensor modeling, paired with recent large‐scale harmonization efforts of well‐established cohorts of aging and AD, such as the Alzheimer's Disease Sequencing Project Phenotype Harmonization Consortium (ADSP‐PHC), provides an unprecedented opportunity to evaluate imaging genetic associations in cohorts enriched with cognitive impaired participants.

Large‐scale genome‐wide association studies (GWASs) have identified and validated novel genetic risk loci for AD.^13^, ^14^, ^15^, ^16^, ^17^, ^18^ However, the precise disease‐associated biological mechanisms for many of these variants remain unknown. By integrating genetic information with FW‐corrected dMRI metrics, we aim to understand how well‐validated AD risk variants affect the microstructure of limbic WM tracts in later life, which are crucial for cognitive processing and memory function and are among the earliest to be impacted in AD progression. For this purpose, we selected genetic variants linked to AD from various GWASs as well as computed polygenic scores (PGS) for AD and analyzed their associations with WM microstructure in limbic tracts derived from non‐Hispanic White aging individuals from seven well‐established harmonized cohorts (*N* = 2614).

## 2 METHODS

### 2.1 Participants

dMRI and genetic data were leveraged from seven cohorts: Alzheimer's Disease Neuroimaging Initiative (ADNI), Biomarkers of Cognitive Decline Among Normal Individuals (BIOCARD), Baltimore Longitudinal Study of Aging (BLSA), National Alzheimer's Coordinating Center (NACC), Religious Orders Study and Rush Memory and Aging Project (ROSMAP), Vanderbilt Memory and Aging Project (VMAP), and the Wisconsin Registry for Alzheimer's Prevention (WRAP). The ADNI project (https://adni.loni.usc.edu) started in 2003 as a public–private initiative, collecting data from cognitively unimpaired (CU) individuals, those with mild cognitive impairment (MCI), and participants diagnosed with AD. The aim was to explore the relationships between serial MRI, positron emission tomography (PET), other biomarkers, clinical and neuropsychological evaluations, and the progression from MCI to early AD.^19^ This study included the data from the ADNI‐Grand Opportunity (GO), ADNI2, and ADNI3 phases. BIOCARD began in 1995 at Johns Hopkins University with the aim of identifying preclinical biomarkers of cognitive decline and predicting future progression to AD in CU middle‐aged individuals. Participants undergo comprehensive longitudinal evaluations, including neuropsychological testing, MRI scans, and collection of blood and cerebrospinal fluid samples.^20^ The BLSA cohort initiated data collection in 1994, focusing on dementia‐free individuals aged 55 to 85 years. In 2009, the cohort was expanded to include participants aged 20 to 85 years, incorporating MRI data collection.^21^ BLSA data can be accessed by submitting a proposal through their website (www.blsa.nih.gov). NACC maintains a centralized data repository for the National Institute on Aging's (NIA's) Alzheimer's Disease Research Centers (ADRC) Program, which currently includes 33 centers and four exploratory centers across the United States.^22^, ^23^, ^24^ The ROS cohort, initiated in 1994, is a continuous longitudinal study collecting clinical and pathological data on aging and AD. Participants are ≥65‐year‐old Catholic nuns, priests, and brothers from various groups across the United States.^25^ MAP, another longitudinal study that began in 1997, recruits cognitively normal participants.^25^ The VMAP cohort began longitudinal data collection in 2012 with the goal of understanding the relationship between vascular health and brain aging enriched in older adults with MCI.^26^ WRAP started data collection in 2001, focusing on middle‐aged adults with a parental history of AD. In 2004, the study expanded to include participants without a parental history of AD. The primary goal of WRAP is to identify early biomarkers and risk factors for AD before clinical symptoms appear.^27^, ^28^

**RESEARCH IN CONTEXT**

1. **Systematic review**: We conducted a comprehensive literature review using PubMed and Web of Science to identify studies that reported genes associated with WM microstructure. While prior research indicated a link between AD risk genes and WM deterioration, no systematic analysis focusing on older adults has been performed.
2. **Interpretation**: Our findings demonstrate that genetic variants associated with AD and polygenic risk for AD significantly influence limbic WM microstructure in later life.
3. **Future directions**: Our study highlights the importance of investigating WM alterations in the context of AD. Future research should aim to expand sample sizes and diversity, which will be instrumental in identifying novel therapeutic targets for AD‐related WM degeneration.

Participants in all cohorts provided written informed consent, and research was conducted in accordance with approved Institutional Review Board protocols. Secondary analysis of these data was approved by the Vanderbilt University Medical School Institutional Review Board. Eight pairs of individuals were related across cohorts and were subsequently removed from the respective cohort with more individuals, namely, BLSA, NACC, and ADNI. To avoid population stratification and underpowered analyses, the study included a total of 2,614 non‐Hispanic White participants aged 50.12 to 100.85 years (mean = 73.66, SD = 9.76), with 42.65% being male. Table 1 provides an overview of the ADNI, BIOCARD, BLSA, NACC, ROSMAP, VMAP, and WRAP cohorts.

**TABLE 1:** Participant characteristics by cohort.

	ADNI	BIOCARD	BLSA	NACC	ROSMAP	VMAP	WRAP	Total
**Number of participants**	491	104	399	659	496	247	218	2614
**Number of females** (%)	236 (48%)	66 (63%)	210 (53%)	371 (56%)	381 (77%)	92 (37%)	143 (66%)	1499 (57%)
**Age** (years)	74.7 (7.6)	73.4 (6.8)	73.2 (9.7)	71.3 (10.5)	81.1 (7.2)	73.7 (7.1)	62.4 (6.1)	73.7 (9.8)
**Education** (years)	16.4 (2.6)	17.5 (2.2)	17.1 (2.4)	15.6 (3.0)	15.8 (3.2)	15.9 (2.7)	16.8 (2.8)	16.2 (2.9)
**Number of *APOE ε4* positive** (%)	184 (37%)	34 (33%)	91 (23%)	281 (43%)	104 (21%)	88 (36%)	68 (31%)	850 (33%)
**Number of base‐line diagnosis** CU (%) / MCI (%) / AD (%)	282 (57%) / 171 (35%) / 38 (8%)	82 (79%) / 20 (19%) / 2 (2%)	395 (99%) / 3 (0.8%) / 1 (0.2%)	416 (63%) / 152 (23%) / 91 (14%)	403 (81%) / 88 (18%) / 5 (1%)	147 (60%) / 99 (40%) / 1 (0.4%)	214 (98%) / 3 (1.4%) / 1 (0.5%)	1939 (74%) / 536 (21%) / 139 (5%)
**Number of cognitively impaired individuals** (%)	235 (48%)	28 (27%)	25 (6%)	251 (38%)	154 (31%)	122 (49%)	4 (2%)	819 (31%)

*Note*: Values denoted as mean (standard deviation) or frequency.

Abbreviations: AD, Alzheimer's Disease; ADNI, Alzheimer's Disease Neuroimaging Initiative; *APOE*, apolipoprotein E; BIOCARD, Predictors of Cognitive Decline Among Normal Individuals; BLSA, Baltimore Longitudinal Study of Aging; CU, cognitively unimpaired; MAP, Memory and Aging Project; MARS, Minority Aging Research Study; MCI, mild cognitive impairment; NACC, National Alzheimer's Coordinating Center; VMAP, Vanderbilt Memory and Aging Project; ROS, Religious Orders Study; WRAP, Wisconsin Registry for Alzheimer's Prevention.

### 2.2 Diffusion MRI acquisition and preprocessing

Across all cohorts, we had 78 different dMRI acquisition protocols – Table S1 provides relevant parameters (e.g., number of directions, b‐values, resolution). dMRI data from all cohorts were processed using the *PreQual* pipeline, which performs motion, susceptibility, and eddy current‐induced distortion and artifact correction while also denoising and imputing slice‐wise signal dropout.^29^, ^30^ Our group used an efficient and scalable parallelization pipeline for the processing.^31^ Manual inspection of imaging sessions was performed by reviewing PDF reports generated by the *PreQual* pipeline. DTIFIT was then used to compute conventional dMRI metrics, including FA_CONV_, AxD_CONV_, MD_CONV_, and RD_CONV_. To account for FW content in each voxel, FW‐corrected metrics were calculated, including FA_FWcorr_, AxD_FWcorr_, MD_Fwcorr_, and RD_FWcorr_, as well as FW.^12^ Symmetric normalization and linear interpolation were performed using the Advanced Normalization Tools (ANTs) package to achieve a standard space representation of these maps by non‐linearly registering the FA_CONV_ map to the FMRIB58_FA atlas.^32^ The resulting warp from the registration was then applied to all other microstructural maps. Individuals with significant age‐regressed outliers (± 5 standard deviations) in WM tract microstructural values were excluded.

### 2.3 WM tractography templates

This study used tractography templates sourced from existing resources,^2^, ^33^, ^34^ which are publicly available in a Zenodo repository.^35^ The focus was on seven WM tracts within the limbic system, specifically the cingulum, fornix, inferior longitudinal fasciculus (ILF), and uncinate fasciculus (UF), as well as the transcallosal tracts of the inferior temporal gyrus (ITG), middle temporal gyrus (MTG), and superior temporal gyrus (STG).

### 2.4 Data harmonization

For the dMRI data, a region of interest (ROI) approach was employed to calculate mean conventional and FW‐corrected dMRI metrics for all tractography templates for each participant. These values were then harmonized using the *Longitudinal ComBat* package in R (version 4.1.0).^36^ Figure S1 depicts a comparison between raw and harmonized FW‐corrected dMRI metrics grouped by diagnosis. This harmonization process used a batch variable that controlled for all imaging batches, as well as several covariates to account for between‐scanner, between‐protocol, and between‐cohort effects. Consistent with our prior work,^1^, ^3^, ^37^ we created a “batch” variable that accounted for various parameters across our cohorts, including scanner name, magnet strength, number of b‐values/b‐vectors, and resolution, optimized to reduce the number of batches while simultaneously accounting for parameters we most anticipated to account for between‐batch heterogeneity. Table S2 shows the batch variables used in the present study. In total, we accounted for 34 unique batching levels. These covariates included mean‐centered age, mean‐centered age squared, education, race/ethnicity, baseline diagnosis, apolipoprotein E (*APOE)* ε4 positivity, *APOE* ε2 positivity, and the interaction of mean‐centered age and cognitive status (CU or cognitively impaired). The cognitive status variable was created by evaluating longitudinal cognitive data in our cohorts, in which an unimpaired cognitive status was given to participants with a constant CU diagnosis, whereas participants with any non‐CU diagnosis, that is, MCI and/or AD at any time point, were given a cognitively impaired status. We enhanced our harmonization approach by applying *Longitudinal ComBat* harmonization to our entire in‐house longitudinal dataset, including 5,144 participants across 10,346 imaging time points. The dataset was then filtered to include only participants with genetic data and further narrowed to the baseline time point for cross‐sectional analysis. The harmonized values were standardized by their respective standard deviations and used in all subsequent statistical analyses. For each participant, we ultimately analyzed FW‐corrected dMRI measures across 48 WM tracts (*N* = 240) at the earliest available age. We then subdivided our data into tracts of interest, which included seven limbic WM tracts. All dMRI measures were scaled and centered for all statistical analyses.

### 2.5 Genetic data quality control and imputation

Genetic data were collected with various genotyping arrays across and within cohorts (ADNI: Illumina Human610‐Quad BeadChip, Illumina HumanOmniExpress BeadChip, Illumina Omni 2.5 M, Illumnia Global Screening Array v2; BIOCARD: Illumina OmniExpress; BLSA: Illumina HumanOmni2.5 BeadChip, Illumina HumanOmniExpress BeadChip; NACC: several different arrays were used to collect genetic data – acquisition of all genetic data is outlined on the NACC website [https://naccdata.org/nacc‐collaborations/partnerships]; ROSMAP: Global Screening Array‐24 version 3.0 BeadChip, Affymetrix GeneChip 6.0, Illumina HumanOmniExpress; VMAP: Illumina HumanOmniExpress; WRAP: Illumina Human610, Illumina OmniExpress).

All genetic raw data underwent the same robust quality control and imputation pipelines.^2^, ^38^, ^39^ Variants that had a genotyping rate less than 95% or a minor allele frequency (MAF) less than 1% or deviated from Hardy–Weinberg equilibrium (*p* < 1 × 10^−6^) were removed. In addition, participants were excluded if genotyping efficiency was poor (missing > 1% of variants), if cryptic relatedness was present (PIHAT > 0.25), or if the reported and genotypic sex were not concordant. Imputation was performed on the University of Michigan Imputation Server using the TOPMed reference panel (hg38) with SHAPEIT phasing.^40^ Data were filtered to exclude variants with low imputation quality (*R* ^2 ^< 0.08), duplicated/multi‐allelic variants, and MAF < 1%. Principal component analysis was conducted, and genetic ancestry outliers were excluded.

### 2.6 Statistical analyses

#### 2.6.1 AD risk variant analysis

To investigate the relationship between AD risk variants and WM microstructure, we compiled a list of 172 SNPs previously associated with AD from various GWASs.^13^, ^14^, ^15^, ^16^, ^17^, ^18^ The list can be found in Table S3. To reduce the burden of multiple testing, this list was further refined using previously published GWAS summary statistics on conventional dMRI metrics, including FA_CONV_, AxD_CONV_, MD_CONV_, and RD_CONV_, derived from UK Biobank data (*N* = 43,802).^41^ These metrics were used to quantify WM in limbic regions, including the cingulum (hippocampus), cingulum (cingulate gyrus), fornix (column and body), fornix–stria terminalis, and UF. Notably, although these regions differ slightly from the tractography templates used in this study, the coverage is similar, and we anticipate that heritability and detectable genetic associations within these regions would be similar. AD risk variants that exhibited at least one significant association (*p*_FDR _< .05, across SNPs and dMRI metrics) with a conventional dMRI metric were retained in the final list of AD risk variants, resulting in 36 AD risk variants for further analysis (Table S4). Results for the significant associations using the UK Biobank WM GWAS summary statistics are displayed in Figure S1. Statistics for all regression models can be found in Table S5.

Next, linear regression models were fitted for each of the 35 FW‐corrected dMRI metrics (five metrics across seven WM tracts), with each of the 36 AD risk variants serving as the predictor of interest. This resulted in a total of 36 × 35 models. The analysis was conducted using Python (version 3.13.0). Visualizations were generated using R (version 4.3.1). Across all analyses, we controlled for age, sex, and the first three ancestral PCs, and *p* values were adjusted across all models using the false discovery rate (FDR) procedure (Benjamini & Hochberg, 1995).

#### 2.6.2 Interaction analysis between AD risk variants and cognitive status

To further investigate the influence of cognitive status, linear regression models were fitted to each dMRI metric (*N* = 35). Predictors included AD risk variant (*N* = 36), AD cognitive status (cognitively impaired individuals vs CU individuals), and the interaction between AD risk variant and cognitive status.

#### 2.6.3 AD polygenic score analysis

To assess the impact of genetic risk for AD on WM microstructure, we calculated a PGS for AD based on the GWAS summary statistics from Marioni et al. (2018).^17^ These specific summary statistics were selected to avoid overlap between participants in the aging cohorts. The AD GWAS summary statistics were prepared using PRS‐CS, a Python‐based tool that estimates posterior SNP effect sizes under continuous shrinkage (CS) priors by integrating GWAS summary statistics with an external linkage disequilibrium (LD) reference panel.^42^ PRS‐CS was also used to calculate PGS for AD without the *APOE* region (chr19, BP 43905796‐45909395). A linear regression model was fitted to each WM tract, with the AD PGS as the primary predictor. To assess the role of cognitive status, a linear regression model was fitted to each WM tract with the predictor AD PGS, cognitive status (cognitively impaired individuals vs CU individuals), and the interaction between AD PGS and cognitive status.

### 2.7 Databases

The genes identified through the statistical analyses were further evaluated using several databases. Specifically, GeneCards (https://www.genecards.org), Agora (https://agora.adknowledgeportal.org), and Open Targets (https://www.opentargets.org) were leveraged. Additionally, a literature search was conducted using PubMed and Web of Science to provide further context and insights into the identified genes.

## 3 RESULTS

### 3.1 AD risk variant associations with WM microstructure: main effects

**FIGURE 1:**
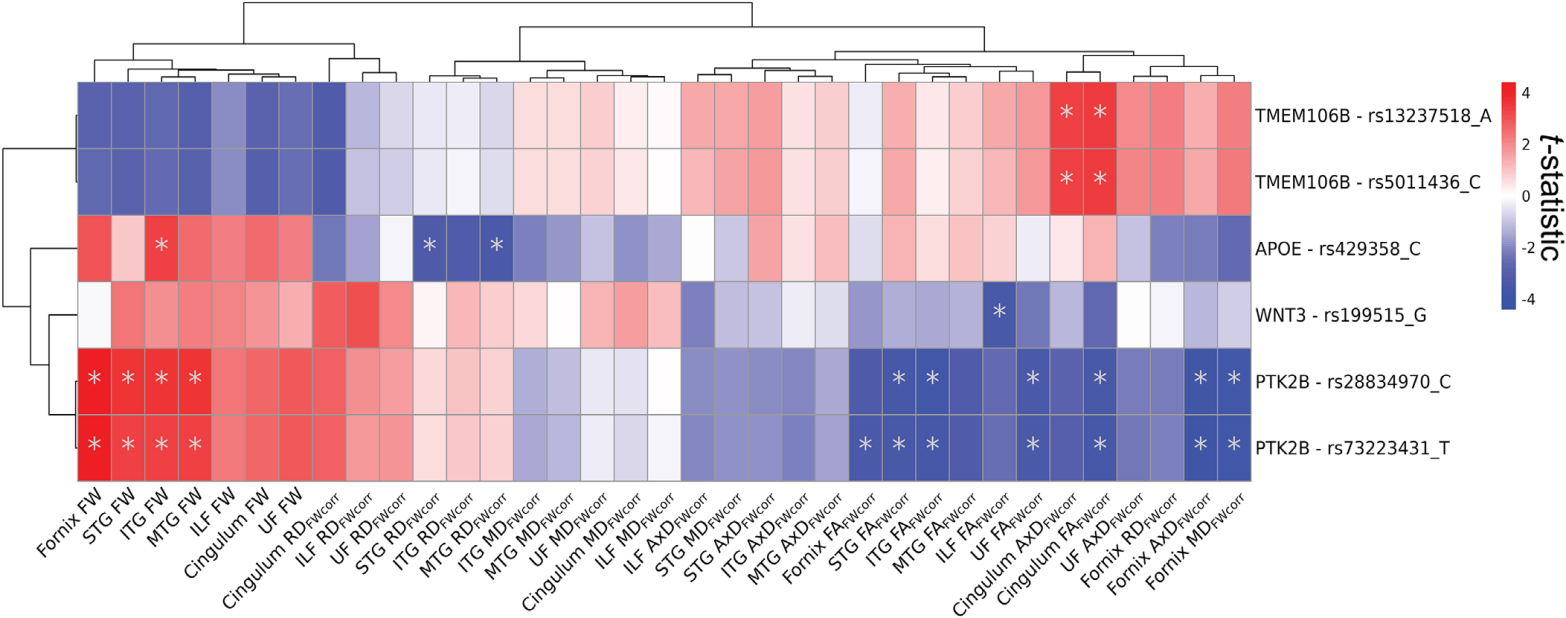
Effects of AD genetic variants on WM microstructure. The figure displays the *t*‐statistics of the main effect AD genetic variant derived from linear regression models fitted separately to each WM microstructure of the limbic tracts measured by FW‐corrected dMRI metrics (*N* = 35). Predictors were 36 genetic variants previously associated with AD and significant in UK Biobank NIDPs. Model: *WM metric* *≈ AD genetic variant + sex + age + PC1 + PC2 + PC3*. The displayed results are filtered for genetic variants that displayed at least one FDR‐significant association (*p*_FDR _< .05) with a dMRI metric. Asterisk indicates *p*_FDR _< .05. Associations were clustered with Euclidean distance approach using pheatmap R package (version 1.0.12). AD, Alzheimer's disease; AxD, axial diffusivity; dMRI, diffusion magnetic resonance imaging; FA, fractional anisotropy; FDR, false discovery rate; FW, free water; ILF, inferior longitudinal fasciculus; ITG, inferior temporal gyrus transcallosal tract; MD, mean diffusivity; MTG, middle temporal gyrus transcallosal tract; NIDP, neuroimaging‐derived phenotype; RD, radial diffusivity; STG, superior temporal gyrus transcallosal tract; WM, white matter.

In our linear models associating AD risk variants with WM microstructure, we identified six variants previously annotated with the genes *TMEM106B*, *PTK2B*, *WNT3*, and *APOE* that were significantly associated with WM microstructure. Figure 1 illustrates the derived *t*‐statistics for models showing at least one significant main effect of AD risk variant (*p*_FDR _< .05) on a dMRI metric. For *TMEM106B*, we found significant positive associations between rs5011436 and both cingulum bundle AxD_FWcorr_ (*β* = 0.099 ± 0.030; *p*_FDR_ = .049) and FA_FWcorr_ (*β* = 0.103 ± 0.030; *p*_FDR_ = .049), as well as between rs13237518 and both cingulum AxD_FWcorr_ (*β* = 0.099 ± 0.030; *p*_FDR_ = .049) and FA_FWcorr_ (*β* = 0.103 ± 0.030; *p*_FDR_ = .049). For these variants, we also found suggestive significance in cingulum FW and RD_FWcorr_, but significance did not survive correction for multiple comparisons. For the variant rs199515, previously annotated to *WNT3*, we found a negative association with ILF FA_FWcorr_ (*β* = −0.123 ± 0.036; *p*_FDR_ = .049).

We found significant positive associations in variants previously annotated to *PTK2B* (i.e., rs28834970, rs73223431) with FW, with the most significant association being between rs28834970 and the fornix (*β* = 0.102 ± 0.023; *p*_FDR_ = .008). These variants were also negatively associated with FA_FWcorr_ in the cingulum (rs28834970: *β* = −0.104 ± 0.031; *p*_FDR_ = .049; rs73223431: *β* = −0.107 ± 0.031; *p*_FDR_ = .049), ITG (rs28834970: *β* = −0.107 ± 0.030; *p*_FDR_ = .049; rs73223431: *β* = −0.105 ± 0.030; *p*_FDR_ = .049), STG (rs28834970: *β* = −0.110 ± 0.031; *p*_FDR_ = .049; rs73223431: *β* = −0.104 ± 0.031; *p*_FDR_ = −.049), UF (rs28834970: *β* = −0.101 ± 0.031; *p*_FDR_ = .049; rs73223431: *β* = −0.101 ± 0.031; *p*_FDR_ = .049), and fornix (rs73223431: *β* = −0.096 ± 0.029; *p*_FDR_ = .049), in addition to fornix AxD_FWcorr_ (rs28834970: *β* = −0.102 ± 0.027; *p*_FDR_ = .049; rs73223431: *β* = −0.105 ± 0.027; *p*_FDR_ = .049) and MD_FWcorr_ (rs28834970: *β* = −0.100 ± 0.027; *p*_FDR_ = .049; rs73223431: *β* = −0.096 ± 0.027; *p*_FDR_ = .049). Finally, the rs429358 variant previously annotated to *APOE* was positively associated with ITG FW (*β* = 0.121 ± 0.037; *p*_FDR_ = .049) but negatively associated with STG RD_FWcorr_ (*β* = −0.137 ± 0.042; *p*_FDR_ = .049) and MTG RD_FWcorr_ (*β* = −0.146 ± 0.044; *p*_FDR_ = .049). Statistics for all regression models can be found in Table S6.

### 3.2 AD risk variant associations with WM microstructure: interactions with cognitive status

**FIGURE 2:**
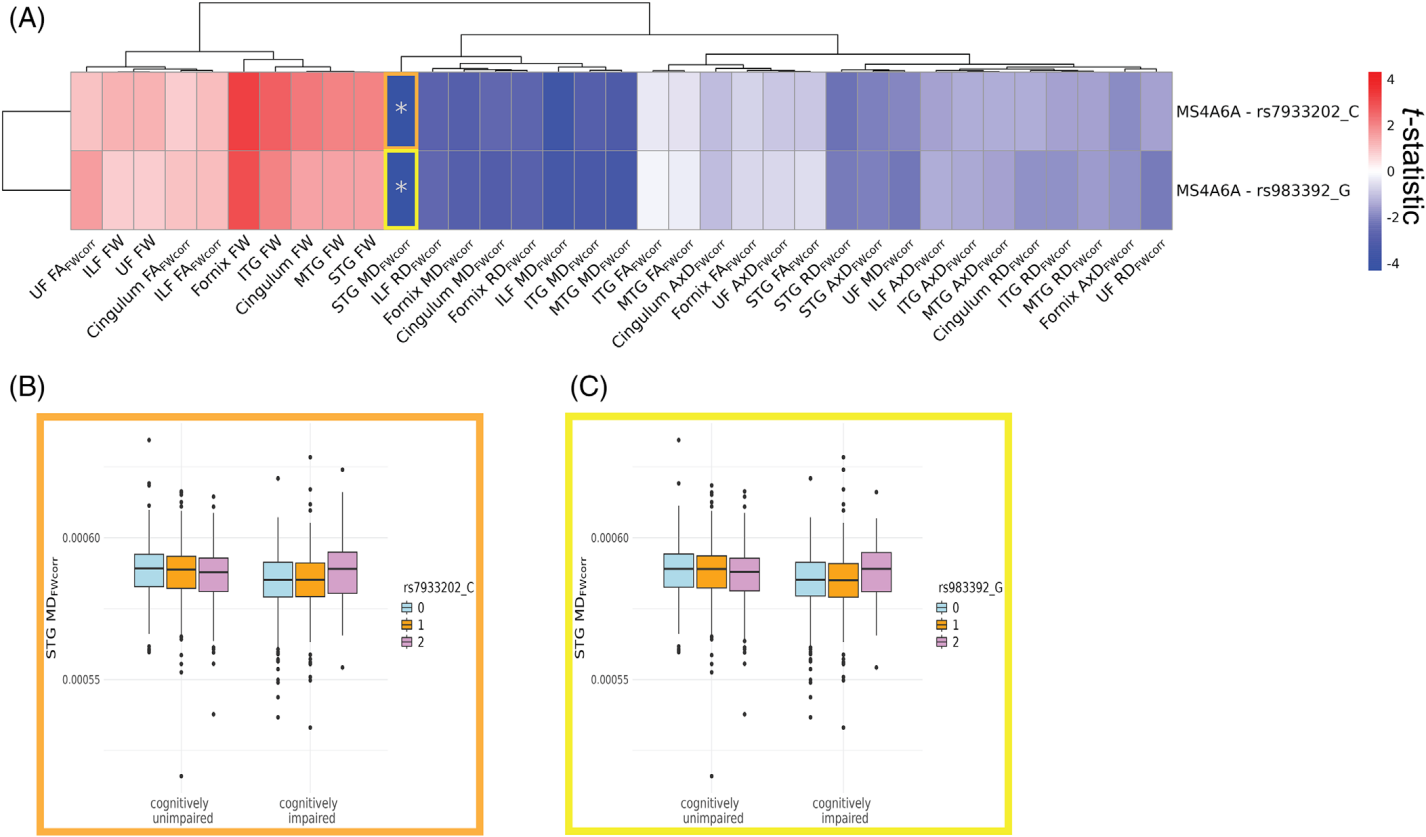
Interaction effects between AD genetic variants and cognitive status on WM microstructure. (A) *T*‐statistics of interaction effect between AD genetic variant and cognitive status derived from linear regression models fitted separately to each WM microstructure of limbic tracts measured by FW‐corrected dMRI metrics (*N* = 35). Model: *WM metric* ≈ AD genetic variant + cognitive status + AD genetic variant * cognitive status + sex + age + PC1 + PC2 + PC3. The displayed results are filtered for genetic variants that displayed at least one FDR‐significant interaction (*p*_FDR_ < .05) with a dMRI metric. Asterisk indicates *p*_FDR _< .05. Associations were clustered with Euclidean distance approach using pheatmap R package (version 1.0.12). (B and C) Exemplary boxplots for STG MD_FWcorr_ and cognitive status, grouped by AD genetic risk variant. AD, Alzheimer's disease; AxD, axial diffusivity; dMRI, diffusion magnetic resonance imaging; FA, fractional anisotropy; FDR, false discovery rate; FW, free water; ILF, inferior longitudinal fasciculus; ITG, inferior temporal gyrus transcallosal tract; MD, mean diffusivity; MTG, middle temporal gyrus transcallosal tract; PC, principal component; RD, radial diffusivity; STG, superior temporal gyrus transcallosal tract; WM, white matter.

When including interactions between AD risk variants and cognitive status, we identified significant negative interaction effects for two variants in *MS4A6A* on STG MD_FWcorr_, including rs983392 (*β* = −0.261 ± 0.063; *p*_FDR_ = .019) and rs7933202 (*β* = −0.274 ± 0.064; *p*_FDR_ = .019) (Figure 2, Table S7). Follow‐up analyses for these two variants stratified for cognitive status showed that both rs7933202 and rs983392 were associated with lower MD_FWcorr_ metrics among CU individuals (rs983392: *β* = 0.170 ± 0.056; *p* = 0.002; rs7933202: *β* = 0.166 ± 0.057; *p* = 0.004), while cognitively impaired individuals exhibited the opposite trend, with the alternative alleles correlating with higher values for these same metrics (rs983392: *β* = −0.091 ± 0.025; *p* = 0.009; rs7933202: *β* = −0.108 ± 0.035; *p* = 0.002).

### 3.3 AD polygenic risk associations with WM microstructure

**FIGURE 3:**
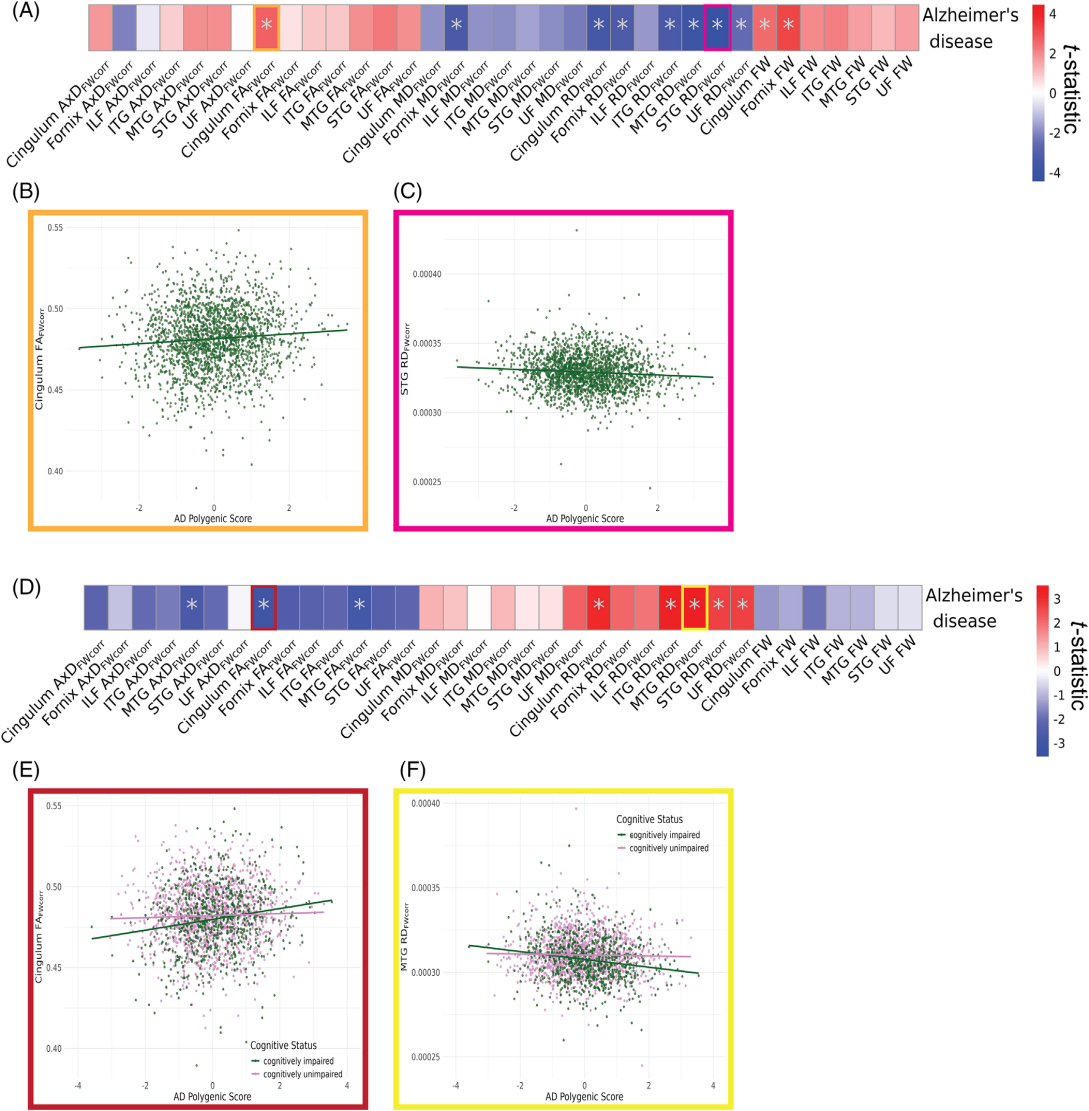
Effects of AD polygenic risk on WM microstructure. (A) *T*‐statistics of main effect AD PGS derived from linear regression models fitted separately to each WM microstructure of limbic tracts measured by FW‐corrected dMRI metrics (*N* = 35). Model: *WM metric* *≈ AD PGS + sex + age + PC1 + PC2 + PC3*. (B and C) show exemplary scatterplots for the main effect AD PGS on (B) cingulum FA_FWcorr_ and (C) STG RD_FWcorr_. (D) *T*‐statistics derived from linear regression models fitted separately to each FW‐corrected dMRI metric model: WM metric ≈ AD PGS + cognitive status + AD PGS * cognitive status + sex + age + PC1 + PC2 + PC3. (E and F) Exemplary scatterplots for interaction effect between AD PGS and cognitive status in (E) cingulum FA_FWcorr_ and (F) MTG RD_FWcorr_. Asterisk indicates *p*_FDR _< .05. AD, Alzheimer's disease; AxD, axial diffusivity; dMRI, diffusion magnetic resonance imaging; FA, fractional anisotropy; FDR, false discovery rate; FW, free water; ILF, inferior longitudinal fasciculus; ITG, inferior temporal gyrus transcallosal tract; MD, mean diffusivity; MTG, middle temporal gyrus transcallosal tract; PC, principal component; PGS, polygenic score; RD, radial diffusivity; STG, superior temporal gyrus transcallosal tract; WM, white matter.

Polygenic risk for AD had several significant associations with dMRI metrics. Specifically, we observed significant positive associations with FW and FA_FWcorr_ measures, with the top association being with the fornix FW (*β* = 0.053 ± 0.016; *p*_FDR_ = .006). We also found several significant negative associations with RD_FWcorr_ and MD_FWcorr_, with the top associations being found in STG RD_FWcorr_ (*β* = −0.100 ± 0.021; *p*_FDR_ = .0003) and fornix MD_FWcorr_ (*β* = −0.060 ± 0.018; *p*_FDR_ = .006) (Figure 3A–C, Table S8). When removing the *APOE* region from the PGS and repeating the analysis, we observed similar effect directions, but none of the associations remained significant after correction for multiple testing (Figure S2, Table S9).

When including an interaction between AD PGS and cognitive status, we found that higher AD PGSs had different effects between cognitively impaired individuals and CU individuals. We observed several positive associations with RD_FWcorr_ of the cingulum (*β* = 0.139 ± 0.045; *p*_FDR_ = .017), ITG (*β* = 0.149 ± 0.045; *p*_FDR_ = .016), MTG (*β* = 0.160 ± 0.045; *p*_FDR_ = .013), STG (*β* = 0.118 ± 0.044; *p*_FDR_ = .035), and UF (*β* = 0.120 ± 0.045; *p*_FDR_ = .035), in addition to negative associations with MTG AxD_FWcorr_ (*β* = −0.120 ± 0.045; *p*_FDR_ = .035), cingulum FA_FWcorr_ (*β* = −0.137 ± 0.044; *p*_FDR_ = .017), and MTG FA_FWcorr_ (*β* = −0.126 ± 0.044; *p*_FDR_ = .028) (Figure 3D–F; Table S10). Follow‐up stratified analyses for cognitive status demonstrated that among cognitively impaired individuals, higher AD PGSs were associated with decreased RD_FWcorr_ but increased AxD_FWcorr_ and FA_FWcorr_ metrics, while CU individuals showed no significant differences (Table S11).

## 4 DISCUSSION

This study investigated the role of AD genetic risk variants in alterations in the limbic WM. By leveraging advanced FW‐corrected WM metrics and integrating genetic data, we identified six variants near the genes *TMEM106B*, *WNT3*, *PTK2B*, and *APOE* as being associated with WM microstructure in a cohort of older adults. The alternative alleles of variants in *TMEM106B* were typically associated with more intact WM microstructure, while the alternative alleles of *PTK2B* and *WNT3* variants were associated with less intact WM microstructure. Our interaction analysis found that cognitive status had distinct dosage‐dependent associations with genetic variants in *MS4A6A*, with the alternative alleles being associated with reductions in MD_FWcorr_ metrics among cognitively impaired individuals, while CU individuals exhibit opposite effects. In our AD PGS analysis, we observed that a higher risk for AD was associated with higher FW and FA_FWcorr_ but lower MD_FWcorr_ and RD_FWcorr_ metrics. After the removal of the *APOE* region, the effect directions remained similar but were not significant. An interaction analysis on AD PGS found that among cognitively impaired individuals, higher polygenic risk for AD was associated with decreased RD_Fwcorr_ but increased AxD_FWcorr_ and FA_FWcorr_ metrics, while CU individuals showed no differences.

When evaluating the biological mechanisms associated with the identified genes in the literature and several databases, we found that the majority were implicated in neurodevelopment (i.e., *WNT3*),^43^, ^44^, ^45^ lipid metabolism (i.e., *APOE*),^5^, ^46^, ^47^ and inflammatory mechanisms (i.e., *TMEM106B*, *PTK2B*, *MS4A6A*).^48^, ^49^, ^50^, ^51^, ^52^, ^53^ All identified genes are expressed in the brain (https://agora.adknowledgeportal.org). Specifically, *TMEM106B*, which has positive effects on WM microstructure in our study, is a glycoprotein expressed in neurons and glial cells that regulate lysosomal trafficking, acidification, and dendrite morphogenesis. Loss of *TMEM106B* exacerbates tau pathology, axonal damage, lipid droplet accumulation, and neurodegeneration in mouse models, suggesting *TMEM106B* has a protective role in neurodegenerative diseases.^48^, ^49^ Furthermore, deficiency in *TMEM106B* leads to reduced microglial survival, proliferation, and activation in response to demyelination, indicating a strong role of *TMEM106B* in immune response and myelination.^50^

In contrast, genetic variants associated with the genes *PTK2B* and *WNT3* indicate risk for harmful WM microstructure alterations in our study. *PTK2B* encodes a cytoplasmic protein tyrosine kinase, which is involved in the regulation of calcium‐induced regulation of ion channels and the activation of the MAP kinase signaling pathway. It is thought to be an important signaling intermediate between neuropeptide‐activated receptors or neurotransmitters that increase calcium flux and the downstream signals that regulate neuronal activity and synaptic plasticity. In addition, *PTK2B* has been implicated in the regulation of inflammatory processes, particularly in microglia activation and migration.^51^ *WNT3* is a member of the Wnt family of signaling proteins and is critical for various developmental processes and cell fate regulation, including neurodevelopmental processes. *WNT3* expression persists in the adult hippocampus and is released by astrocytes to regulate adult neurogenesis,^43^ and reduced levels of paracrine *WNT3* factors during aging are associated with impaired neurogenesis in the adult hippocampus,^43^, ^44^, ^45^ which potentially extends to reduced WM microstructure.

This study identified a significant effect of *APOE* (the strongest genetic driver of sporadic AD) on WM microstructure. Specifically, the alternative allele of variant rs429358 was positively associated with ITG FW and negatively associated with STG RD_FWcorr_ and MTG RD_FWcorr_. Previous studies reported widespread effects on WM when comparing *APOE* ε4 carriers (homozygotes and heterozygotes) to non‐carriers, characterized by reduced FA_CONV_ alongside increased AxD_CONV_, MD_CONV_, and RD_CONV_.^5^ In the brain, *APOE* is a key player in cholesterol transport and redistribution by binding to lipoprotein particles and delivering cholesterol to neurons. Cholesterol is essential for membrane repair, synapse formation, and myelination. However, the *APOE* ε4 isoform is less efficient at transporting lipids, which can impair myelination and the maintenance of WM microstructure.^46^, ^47^

When investigating the interaction between AD risk variants and cognitive status, we identified a different subset of variants in the gene *MS4A6A*. This suggests that the genetic factors influencing the initial changes in WM microstructure might differ from those that affect the progression to cognitive impairment. The gene *MS4A6A*, a member of the membrane‐spanning four‐domain subfamily A (*MS4A*) gene family, has been implicated in various pathological conditions including neurodegenerative diseases and glioblastoma by modulating immune responses. It encodes a protein that plays a critical role in regulating immune signaling pathways, particularly in macrophages and microglia, which are important for maintaining homeostasis in the central nervous system and modulating inflammatory pathways.^52^, ^53^

These results indicate that AD‐relevant biological mechanisms are associated with WM changes in aging. Further research should evaluate the incorporation of WM neurodegeneration measures into the AT(N) framework.^54^ All of the identified AD risk variants were present with similar effect directions in GWAS summary statistics on WM derived from UK Biobank data,^41^ which predominantly include younger individuals compared to our cohort. This underscores the early influence of these AD risk variants on altering WM microstructure, potentially manifesting years before the onset of clinical symptoms. Notably, only ∼5% of our cohort had a clinical AD diagnosis at the time of imaging. In a subset (*N* = 678) with T1‐weighted hippocampal atrophy data, ∼26% showed overt atrophy (volume ≤ 6723 mm^3^).^55^ Despite these low proportions, our findings suggest that WM alterations may serve as a potential early signal of AD.

Investigating the relationship between AD genetic risk and WM microstructure in well‐established cohorts of older individuals allows us to better understand the effect of AD mechanisms on WM. Additionally, the use of FW‐corrected dMRI metrics captures WM microstructure with higher biological accuracy. However, this study has several limitations. This analysis only included non‐Hispanic White individuals with European ancestry. While this approach helps to avoid population stratification, it also limits the generalizability of the results. The sample size is not ideal for genetic analysis. In future research, we will replicate our findings in more diverse cohorts to ensure broader generalizability and robustness of the presented results. Furthermore, all our analyses are cross‐sectional and correlational in nature and do not indicate causality. While the use of single‐shell dMRI data allowed for the inclusion of a larger cohort, multi‐shell approaches, such as NODDI,^56^ provide the advantage of deconvolving the diffusion signal into distinct tissue compartments, offering a more detailed understanding of microstructural features. Our team is currently exploring novel deep learning techniques aimed at improving the accuracy of FW estimation using single‐shell data. Once validated, these methods will be incorporated into our future studies. Additionally, future research using multi‐shell diffusion models could yield deeper insights into the interplay between neuroinflammation and its subtle effects on the diffusion weighted imaging (DWI) signal, potentially enabling the quantification of neuroinflammation. Continued investigation is essential to unravel the complex relationships between FW‐corrected dMRI metrics, AD risk, and WM microstructure.

We found potential links between genetic variants associated with AD and limbic WM microstructure in later life, crucial for cognitive function and memory. The genes identified were related to neurodevelopment (i.e., *WNT3*), lipid metabolism (i.e., *APOE*), and inflammatory mechanisms (i.e., *TMEM106B*, *PTK2B*, *MS4A6A*). These findings indicate that genetic factors contributing to AD may drive early alterations in WM microstructure, observed in a cohort where only ∼5% had received an AD diagnosis at the time of imaging.

## CONFLICT OF INTEREST STATEMENT

S.C.J. has served on advisory boards for Enigma Biomedical and ALZPath in the past two years. A.J.S. receives support from multiple NIH grants (P30 AG010133, P30 AG072976, R01 AG019771, R01 AG057739, U19 AG024904, R01 LM013463, R01 AG068193, T32 AG071444, U01 AG068057, U01 AG072177, U19 AG074879, and U24 AG074855). He has also received support from Avid Radiopharmaceuticals, a subsidiary of Eli Lilly (in kind contribution of PET tracer precursor) and participated in Scientific Advisory Boards (Bayer Oncology, Eisai, Novo Nordisk, and Siemens Medical Solutions USA, Inc.) and an Observational Study Monitoring Board (MESA, NIH NHLBI), as well as External Advisory Committees for multiple NIA grants. He also serves as Editor‐in‐Chief of Brain Imaging and Behavior, a Springer‐Nature Journal. Author disclosures are available in the Supporting Information.

## CONSENT STATEMENT

All participants provided informed consent in their respective cohort studies.

## Supporting information



SUPPLEMENTARY FIGURE S1. Raw and harmonized FW‐corrected dMRI metrics by diagnosis. *Note*: This figure compares harmonized and raw FW‐corrected dMRI metrics for each limbic tract. Abbreviations: AD, Alzheimer's disease; AxD, axial diffusivity; CU, cognitively unimpaired; dMRI, diffusion magnetic resonance imaging; FA, fractional anisotropy; FW, free water; ILF, inferior longitudinal fasciculus; ITG, inferior temporal gyrus transcallosal tract; MCI, mild cognitive impairment; MD, mean diffusivity; MTG, middle temporal gyrus transcallosal tract; RD, radial diffusivity; STG, superior temporal gyrus transcallosal tract; UF, uncinate fasciculus.

SUPPLEMENTARY FIGURE S2. Effects of AD genetic risk variants on WM microstructure measured by conventional dMRI metrics in UK Biobank. *Note*: This figure displays the z‐statistics of AD genetic variants derived from a GWAS on WM, measured by conventional dMRI metrics (*N* = 20), using UK Biobank data (Zhao et al., 2021) and including 172 genetic variants previously associated with AD. The displayed results are filtered for genetic variants that displayed at least one FDR‐significant association with a dMRI metric (p_FDR _< .05, multiple testing correction across 20 limbic dMRI metrics and 172 genetic variants). Asterisk indicates p_FDR _< .05. The associations were clustered with the Euclidean distance approach using the pheatmap R package (version 1.0.12). Abbreviations: AD, Alzheimer's disease; AxD, axial diffusivity; CONV, conventional; dMRI, diffusion magnetic resonance imaging; FA, fractional anisotropy; GWAS, genome‐wide association study; MD, mean diffusivity; RD, radial diffusivity; UF, uncinate fasciculus; WM, white matter.

SUPPLEMENTARY FIGURE S3. Effects of AD polygenic risk on WM microstructure when *APOE* region is removed. (A) *T*‐statistics of main effect AD PGS with removed *APOE* region derived from linear regression models fitted separately to each WM microstructure of limbic tracts measured by FW‐corrected dMRI metrics (*N* = 35). Model: *WM metric ≈ AD PGS without APOE + sex + age + PC1 + PC2 + PC3*. No association remained significant after multiple testing correction. Abbreviations: AD, Alzheimer's disease; AxD, axial diffusivity; FA, fractional anisotropy; FW, free water; ILF, inferior longitudinal fasciculus; ITG, inferior temporal gyrus transcallosal tract; MD, mean diffusivity; MRI, magnetic resonance imaging; MTG, middle temporal gyrus transcallosal tract; PC, principal component; PGS, polygenic score; RD, radial diffusivity; STG, superior temporal gyrus transcallosal tract; UF, uncincate fasciculus; WM, white matter.

Supporting Information

Supporting Information
