# Supplementary figures and images for: The effect of Alzheimer's disease genetic factors on limbic white matter microstructure
Source: Alzheimers Dement. 2025 Apr 12;21(4):e70130. doi: 10.1002/alz.70130 (PMC11992597; doi:10.1002/alz.70130)

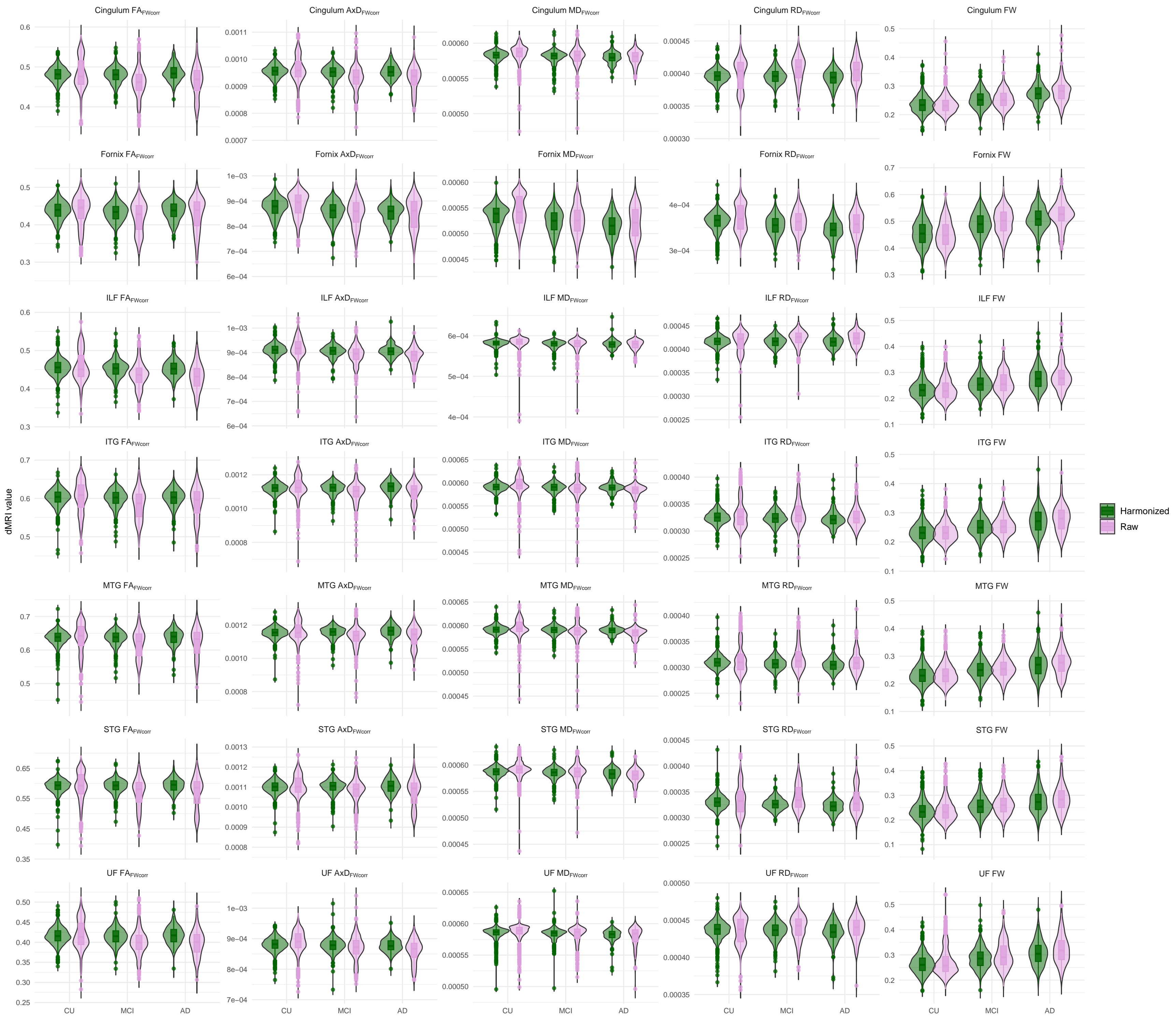

Supplement: Supplementary file 1 — SUPPLEMENTARY FIGURE S1. Raw and harmonized FW‐corrected dMRI metrics by diagnosis. Note: This figure compares harmonized and raw FW‐corrected dMRI metrics for each limbic tract. Abbreviations: AD, Alzheimer's disease; AxD, axial diffusivity; CU, cognitively unimpaired; dMRI, diffusion magnetic resonance imaging; FA, fractional anisotropy; FW, free water; ILF, inferior longitudinal fasciculus; ITG, inferior temporal gyrus transcallosal tract; MCI, mild cognitive impairment; MD, mean diffusivity; MTG, middle temporal gyrus transcallosal tract; RD, radial diffusivity; STG, superior temporal gyrus transcallosal tract; UF, uncinate fasciculus. [file ALZ-21-e70130-s001.pdf]
